# Adenocarcinoma of the appendix presenting as bilateral ureteric obstruction

**DOI:** 10.1186/1477-7819-6-23

**Published:** 2008-02-21

**Authors:** Kamran Ahmed, Robiol Hoque, Sherif El-Tawil, Mohammad S Khan, Mark L George

**Affiliations:** 1 Department of General Surgery, St Thomas' Hospital, London, UK; 2 Department of Urology, Guy's Hospital, London, UK

## Abstract

**Background:**

Adenocarcinoma of the vermiform appendix is a rare neoplasm of the gastrointestinal tract. Presentation mimics acute appendicitis, but right iliac fossa mass and intestinal obstruction have also been reported. These presentations reflect various stages of a locally expanding tumour causing luminal obstruction of appendix. The investigation and subsequent management with a review of the literature is presented.

**Case presentation:**

We report a case of appendicular adenocarcinoma found unexpectedly in a 43 year old male who presented with urinary symptoms. Cystoscopy and uretero-renoscopy showed normal bladder but external compression of the ureters and therefore bilateral stents were inserted. CT scan showed a caecal mass. After colonoscopy, that showed external compression, and diagnostic laparoscopy the patient underwent right hemicolectomy. Histopathology revealed well differentiated adenocarcinoma with signet ring morphology with multiple lymph node involvement. The patient was referred for chemotherapy where he received infusional 5 fluorouracil but died 7 months after surgery.

**Conclusion:**

Patients with atypical manifestations related to right lower abdominal quadrant should be thoroughly investigated with an open mind. Every attempt should be made to make a precise diagnosis through all the available means to direct the treatment along correct lines.

## Background

The appendix is an uncommon site of gastrointestinal malignancy. Presentation mimics acute appendicitis, but right iliac fossa mass and intestinal obstruction have been reported. These presentations reflect various stages of a locally expanding tumour causing luminal obstruction of appendix.

There are other clinical presentations and here we report a case of appendicular signet ring cell adenocarcinoma found unexpectedly in a patient who presented to the urologists with urinary symptoms.

## Case presentation

A 43 years old male presented to the emergency department with a two week history of right lower quadrant pain radiating to the right testis. Baseline blood tests were normal apart from creatinine of 227 (umol/l) and blood in the urine. An intravenous urogram showed bilateral ureteric obstruction with a standing column of contrast in the ureters extending up to the lower one third of the ureters. The patient was transferred to a specialist unit. Re-examination revealed a right iliac fossa mass and a clinically frozen pelvis on digital rectal examination. Cystoscopy and uretero-renoscopy showed normal bladder but external compression of the ureters and therefore bilateral stents were inserted.

**Figure 1:**
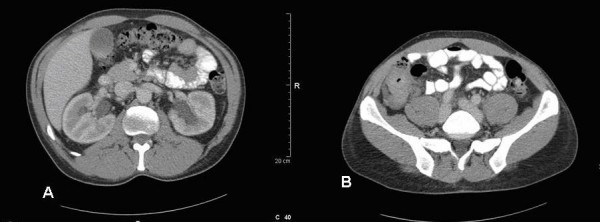
(a&b) – CT Scan: Bilateral hydronephrosis with associated caecal mass (arrow showing hydronephrosis).

**Figure 2:**
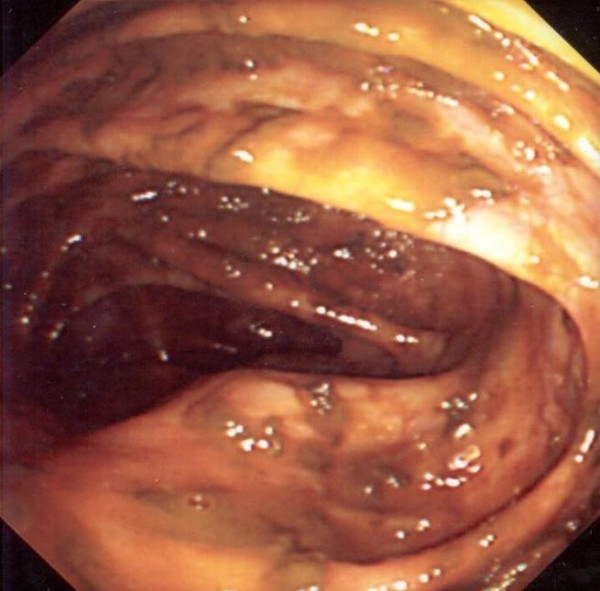
Colonoscopy: Extrinsic compression of caecal pole (arrow showing caecal bulge in the caecal wall).

CT scan (Figure 1) showed a 5 × 4 cm caecal mass with no peritoneal or distal metastatic spread. The patient underwent colonoscopy (Figure 2) which showed extrinsic compression of the caecum, but with no intrinsic lesion.

**Figure 3:**
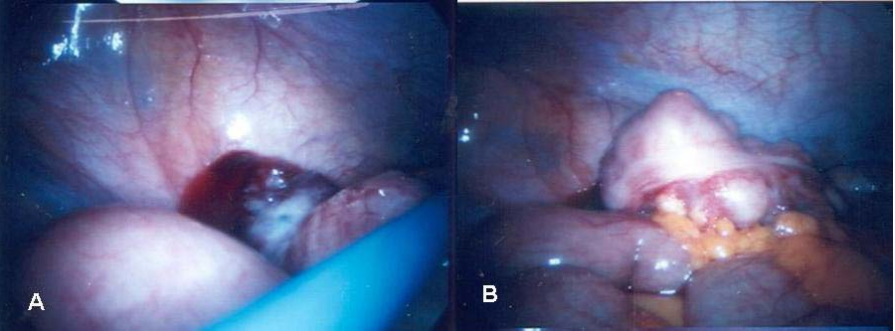
(a&b) Laparoscopy: appendicular tumour.

**Figure 4:**
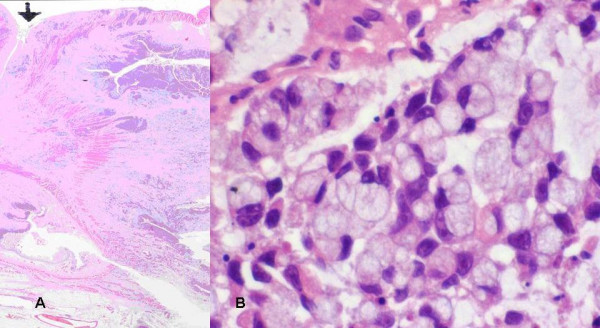
(a&b) Histopathology (H&E stain): Tumour invading bowel wall, breaching serosal surface. Appearance of signet cells. (adenocarcinoma).

A diagnostic laparoscopy (Figure 3) was performed which confirmed a tumour of appendix with biopsies showing poorly differentiated adenocarcinoma. Due to on going pain the patient underwent a laparotomy. The pelvis was frozen secondary to peritoneal disease and a right hemicolectomy was performed. Pathology showed a poorly differentiated adenocarcinoma (Figure 4) with signet ring morphology with multiple lymph node involvement (16/28). The patient was discharged on the third post operative day and referred for chemotherapy. He received infusional 5 fluorouracil but died 7 months after surgery.

## Discussion

Adenocarcinoma of the vermiform appendix is a rare neoplasm of the gastrointestinal tract with an incidence of about 0.01–0.2% [1]. Only about 250 cases of primary adenocarcinoma of appendix have been described since Berger first recognized the neoplasm in 1882.

International Classification of Diseases for Oncology (ICD-O) groups divides the adenocarcinoma of appendix into three categories: colonic, mucinous and signet ring cell adenocarcinoma with a mean age of 60 years (Range 17–90) at diagnosis [2,3] (Table 1).

**Table 1:** Characteristics of patients presenting with appendiceal adenocarcinoma ^3^

**Characteristics**	**Mucinous adenocarcinoma**	**Colonic type adnocarcinoma**	**Signet ring cell carcinoma**
**Age at diagnosis**	60 (Range 17–99)	62 (Range 19–98)	58 (Range 25–90)

**Gender**	Male 49%	Male 60%	Male 46%
	Female 51%	Female 40%	Female 54%
**Race**	White 89%	White 80%%	White 93%
	Black 6%	Black 13%	Black 1%
	Other 5%	Other 7%	Other 4%

Most of the patients present as acute appendicits (37%), frequently with an appendiceal abscess [4]. Rarely is the diagnosis made preoperatively. Unusual presentations have been reported before such as chronic renal failure and a right renal mass and others have reported appendiceal carcinoma presenting as primary ovarian tumour [5,6].

Primary signet cell carcinoma of appendix is an extremely rare entity and is notorious for its spread to adjacent organs (76%) at presentation compared with mucinous (63%) and colonic type (37%) cancers [3]. Survival in this type of tumour group is significantly worse than the other appendiceal carcinoma and some authors stress that this type should be considered a separate type of appendiceal malignancy because of its poor prognosis [3,7].

Right hemicolectomy is considered to be the treatment of choice for all lesions with invasion beyond the mucosa. For in situ carcinoma some authors suggest there is no survival advantage in performing a right hemicolectomy over appendicectomy alone. Varisco *et al*, in meta-analysis involving 100 patients, supported the use of appendicectomy alone in localized cases of adenocarcinoids of the appendix with low tumour histology with no caecal involvement [8]. The role and safety of laparoscopic appendicectomy for management of incidentally discovered appendiceal tumours has not yet been established. Laparoscopic approach has slightly higher rate of inadequate resection. However, it is not associated with a significantly worse patient prognosis than open appendicectomy. The treatment options for metastatic disease include chemotherapy alone, hyperthermic intraoperative intraperitoneal chemotherapy, radical surgery with peritonectomy and combination of treatments. In our case laparotomy also allowed assessment of the pelvis to see if the patient needs peritonectomy and intraoperative chemotherapy, but the pelvis was frozen and therefore this option was not pursued. The treatment for metastatic disease is standard post-operative adjuvant chemotherapy. For management of metastatic peritoneal disease hperthermic intraperitoneal chemotherapy or peritonectomy can be considered in appropriate centers [9,10]. The overall 5-year survival rate for appendicular adenocarcinoma reported by Park *et al *is 20.5% [4].

Studies have reported urological involvement due to direct invasion of tumour into the bladder [11,12]. Previously unilateral ureteral obstruction due to appendiceal carcinoma has been reported by a few authors [13,14]. A case of urinoma formation due to extravasation of urine secondary to ureteral obstruction by metastatic squamous cell carcinoma of the appendix has been described in literature [15]. Risher *et al *reported an incidental finding of calcified mucocele of appendix that was discovered during evaluation of ureteral obstruction [16]. The above mentioned case was unique in a sense that the patient presented with bilateral ureteric obstruction due to the metastatic spread of tumour resulting in a frozen pelvis. Adenocarcinoma of appendix is rare and often presents at an advanced stage. Despite surgery and adjuvant treatment, the prognosis remains poor.

## Conclusion

**Figure 5:**
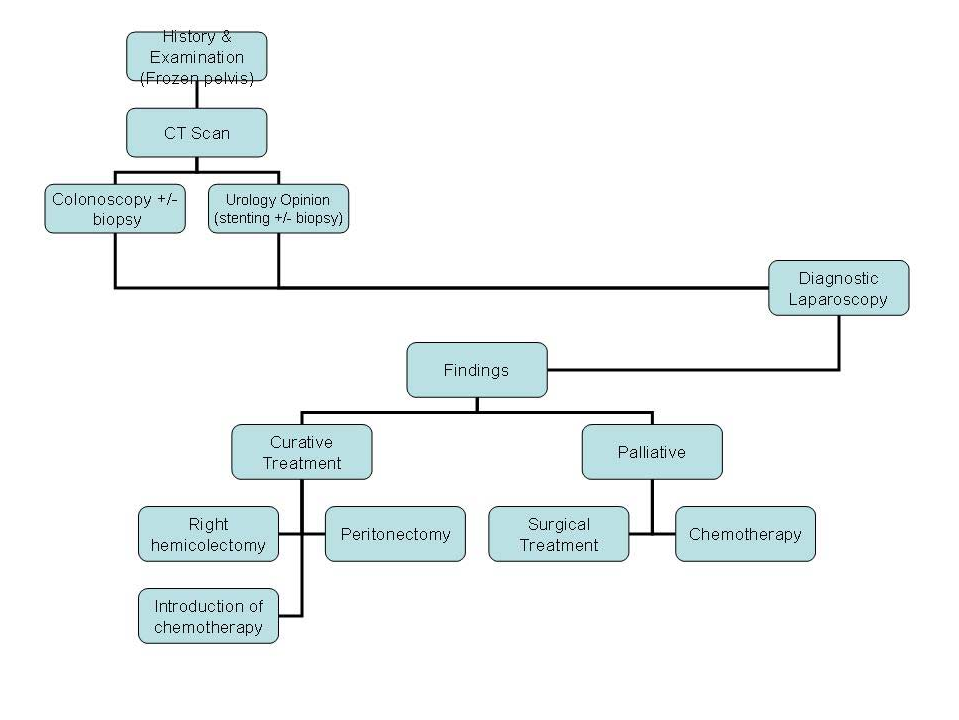
Diagnostic and Management Approach.

Appendicular lesions, both inflammatory and neoplastic, are notorious for atypical presentation. It is thus not surprising that the rate of this misdiagnosis is quite high particularly if solely based on clinical grounds. In conclusion, patients with atypical manifestations related to right lower abdominal quadrant should be thoroughly investigated with an open mind (Figure 5). Every attempt should be made to make a precise diagnosis through all the available means to direct the treatment along correct lines.

## Competing interests

The author(s) declare that they have no competing interests.

## Authors' contributions

KA carried out the design of the study, acquired patient's records, and drafted the manuscript. RH participated in acquisition of data. SET participated in acquisition of data. MSK participated in acquisition of data and added his opinion about the urological aspect of this study. MG (Senior Author) carried out the design of the study, coordinated the study, and drafted the manuscript. All authors read and approved final manuscript.

## References

[B1] Rassu PC, Cassinelli G, Ronzitti F, Bronzino P, Stanizzi T, Casaccia M (2002). Primary adenocarcinoma of the appendix. Case report and review of the literature. Minerva Chir.

[B2] Percy C, Van Holten, Muir C, editors (1990). International Classification of Diseases for Oncology (ICD-O).

[B3] McCusker ME, Cote TR, Clegg LX, Sobin LH (2002). Primary malignant neoplasms of the appendix: a population-based study from the surveillance, epidemiology and end-results program, 1973–1998. Cancer.

[B4] Park IJ, Yu CS, Kim HC, Kim JC (2004). Clinical features and prognostic factors in primary adenocarcinoma of the appendix. Korean J Gastroenterol.

[B5] Parsons JK, Freeswick PD, Jarrett TW (2004). Appendiceal cystadenoma mimicking a cystic renal mass. Urology.

[B6] Liapis A, Michailidis E, Bakas P, Kondi-Pafiti A, Creatasas G (2004). Mucinous tumours of the appendix presenting as primary tumours of the ovary. Report of two cases. Eur J Gynaecol Oncol.

[B7] Thomas RM, Sobin LH (1995). Gastrointestinal cancer. Cancer.

[B8] Varisco B, McAlvin B, Dias J, Franga D (2004). Adenocarcinoid of the appendix: is right hemicolectomy necessary? A meta-analysis of retrospective chart reviews. Am Surg.

[B9] Smejkal P, Pazdro A, Smejkal M, Pafko P, Frantlova M (2005). The cystadenocarcinoma of the appendix. Rozhl Chir.

[B10] Vaira M, Scuderi S, Costamagna, Barone R, Aghemo B, Mioli PR, De Simone M (2001). Cytoreductive surgery and intraperitoneal hyperthermic antiblastic therapy (HAPP) in peritoneal carcinomatosis. Minerva Chir.

[B11] Mori N, Noma M, Hara T, Yamaguchi S, Shibata K, Ishii T, Adachi S (2002). A case of mucinous cystadenocarcinoma of the appendix penetrating the urinary bladder. Hinyokika Kiyo.

[B12] Richie JP (1977). Primary adenocarcinoma of the appendix masquerading as a bladder tumour. Arch Surg.

[B13] VAira M, Scuderi S, Costamagna D, Barone R, Aghemo B, Mioli PR, De Simone M (2001). [Cytoreductive surgery and intraperitoneal hyperthermic-antiblastic therapy (HAPP) in peritoneal carcinomatosis]. Minerva Chir.

[B14] Katsuno G, Kagawa S, Kokudo Y, Muraoka A, Tatemoto A, Sone Y, Tsumura M, Tsuruno M, Mizobuchi K (2005). Ureteral metastasis from appendiceal cancer: report of a case. Surg Today.

[B15] Angulo JC, Lopez JI, Lopez-Arregui E, Flores N (1993). Urinoma formation secondary to ureteral obstruction by metastatic squamous cell carcinoma of the appendix. Case report. Tumori.

[B16] Risher WH, Ray JE, Hicks TC (1991). Calcified mucocele of the appendix presenting as ureteral obstruction. J La State Med Soc.

